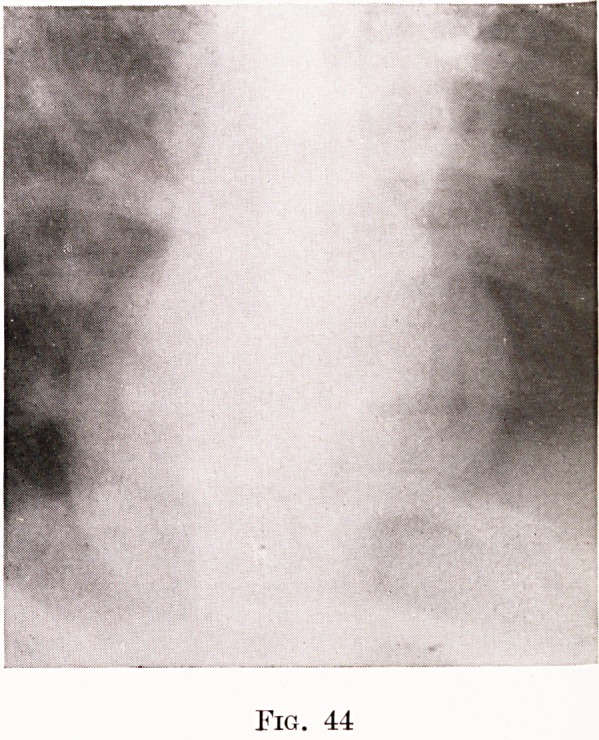# Foreign Bodies in the Gullet

**Published:** 1929

**Authors:** E. Watson-Williams

**Affiliations:** Surgeon-in-charge Ear, Nose and Throat Department, Bristol Royal Infirmary


### The Bristol Medico-Chirurgical Journal

*"Scire est nescire, nisi id me
Scire alius sciret."*

SUMMER, 1929.

### FOREIGN BODIES IN THE GULLET.

BY

E. Watson-Williams, M.C., Ch.M., F.R.C.S.,
*Surgeon-in-charge Ear, Nose and Throat Department,
Bristol Royal Infirmary.*

---

The treatment of patients with foreign bodies impacted in the gullet is a branch of surgery that has in recent years undergone a complete change. Although the œsophagus and stomach were examined in the living subject by means of a tube as far back as the middle of the eighteenth century, clinical application of this method has been developed only during the twentieth. Before that development the means of dealing with such accidents were distinctly primitive. The obstructing mass was pushed down or hooked up by a blind instrumentation. If this failed, an external œsophagotomy in the neck was necessary. Both procedures were fraught with considerable risk, and accompanied by a serious mortality. One must admire the ingenuity of the expanding probang and the hinged coin-catcher. But in so delicate a tube as the œsophagus the use of such instruments, gentle and skilful though it were, carried a real danger of damage; not solely from the instrument itself, but still more from the sharp edges or points of the foreign body. In a civilized community these appliances have now only one place—the museum.

The cardinal principle of treatment is the avoidance of all blind methods, every step being conducted under direct vision. The patient or parent generally knows what the foreign body is, and how long it has been present. Often, too, we can get some idea of where it is lodged. It must, however, be remembered that babies swallow a strange assortment of objects, and the possibility of a foreign body should not be overlooked in the case of a small patient who has suddenly developed dysphagia. The actual degree of this symptom varies much. A smooth, flat, foreign body may cause little disturbance; more commonly fluids only can be taken. In a few cases nothing at all can be swallowed, owing to the size of the foreign body or to reflex spasm; while if the obstruction is low down food is taken but immediately returned. Difficulty in swallowing is not always due to the actual presence of a foreign body. Damage inflicted by its passage, or during attempts to dislodge it, may from spasm increase the "feeling that there is something there."

Pain indicates irritation or actual laceration of the œsophagus. With slight damage it occurs only on swallowing, and fluids can usually be taken. I have records of six cases of this nature in which the laceration was verified by œsophagoscopy. In two cases it had been caused by a probang, in one by thrusting down a sweet (? sharp edge or finger-nail), in three by the passage of the foreign body itself. For example, a woman who said she had swallowed a piece of glass in preserved fruit was found to have a deep laceration of the mucosa extending 2 cm. down from the lower border of the cricoid. In such cases a disinfectant mouthwash is used at frequent intervals, and formalin lozenges can be sucked. Food should be fluid, sterile and iced, and if necessary half a drachm of orthoform powder may be given twenty minutes before feeding. Bismuth should follow a meal :—

| | | | |
|---|---|---|---|
| ℞. | Bismuth. Oxycarb. | .. | gr. xx. |
| | Lactos. .. .. | .. | gr. xl. |

A powder to be placed on the tongue and swallowed dry.

Where more serious damage has been inflicted there may be complete inability to swallow, attempts increasing the pain which does not cease in the intervals. Some fever is usual, and in a bad case the breath is fetid. Reflex salivation adds materially to the discomfort, and these patients should be nursed prone, the head to one side with only a small pillow under the occiput. In this position saliva can dribble from the mouth, instead of overflowing into the trachea and rendering rest impossible. Proctoclysis may be needed for two or three days until a fluid diet is possible. Three of my patients have needed this treatment. A still more serious condition was found in another, who said she had swallowed a plum-stone. A probang had been passed, without retrieving it, but from that time she could swallow nothing. I found that immediately below the œsophageal entrance the mucosa had been stripped from almost the whole circumference over a distance of just over 2 cm., and hung in the lumen like an œdematous polypus. Gastrostomy was performed: after three weeks she was able to take fluids and made a complete recovery.

In none of the above cases was any foreign body seen. Two further cases are described below. In one a safety-pin penetrated the wall of the hypopharynx; the patient recovered. In the second a sharp splinter of bone had been made to pierce the œsophagus, with fatal results.

At the other end of the scale are those patients whose only evidence of swallowing is the sudden disappearance of an object held in the mouth, a pin or denture. It is curious how often a missing denture is found in the grate! A skiagram should always immediately precede any operative search for the foreign body—immediately, as these bodies sometimes change their position. Before resorting to this we have, of course, examined the pharynx and larynx with the aid of a mirror. Quite often this method suffices to locate an object that the patient thought farther down; and we must not forget that a body in the neighbourhood of the Eustachian orifice may be "felt" along the border of the thyroid cartilage. A skiagram will certainly show a denture, a needle, or any but the most minute fragment of metal. Figures 41 and 42 show the appearance of a coin and a toy watch. Glass, celluloid, fish-bones, meat and possibly small pieces of wood or bone may escape detection, though even with these an opaque meal may afford some help. One can recall the case of a small baby that was subjected to œsophagoscopy because he had "swallowed" a safety-pin, which a subsequent skiagram showed in the nose! In a very similar case I was myself invited to remove from the gullet of a baby three weeks old a safety-pin that he had "swallowed." The skiagram revealed it in the naso-pharynx, where it could not be seen from the mouth and was felt only with considerable difficulty. Once located its removal was easy. (Figures 37 and 43.) On another occasion I was asked to retrieve by means of the œsophagoscope a needle that the skiagram disclosed in the cæcum.

When we have thus located the foreign body, or when we feel, despite negative skiagraphy, that it is reasonably probable that there is one in the gullet, we proceed to examine this region by means of an œsophagoscope. The latter is a metal tube of size appropriate to the age of the patient, with a small electric light at the distal end. I use it without a handle, as being more delicate in manœuvre. It is passed down with great gentleness, no force at all being permissible; and the œsophagus is closely examined during the whole movement, to avoid "over-riding." The need for care cannot be over-emphasized; even an object so blunt as a farthing has been made to penetrate the thin wall of the œsophagus. I prefer a general anæsthetic for this operation, except with nurslings. It is necessary with children, and few adults even can tolerate the presence of the tube for many minutes. It is extremely important that everything should proceed smoothly and without hurry or fuss. Coins are, as a rule, easily removed, though even with these one may regret "demonstrating" instead of removing the coin as soon as found. The appearance of a half-penny when viewed through the œsophagoscope is shown in Figure 22. With bulky objects, needing to be divided with shears, or bodies having hooks or spikes that require disimpaction, a problem of real difficulty may occur. Finally, special instruments may be called for, such as the "closer for safety-pin point upward," illustrated in Figure 45.

FIGURE 45.
*Safety-pin closer, showing method of use.*

A short description is appended of the fifty-three objects in this series, so far as it is interesting (or recorded). Measurements are in each case from the incisor teeth to the nearest part of the foreign body. The youngest patient was three weeks old, the oldest 73 years, and with the one exception mentioned all made a good recovery.

*Coins.* Twenty-four Cases.

*Sixpence.*

Age 1. Coin three days in throat, can take fluids only. Coin found at 10 cm. from teeth and removed. (Fig. 19.)

*Penny.* Two cases.

Age 11. A choir-boy, holding a penny for the collection in his hand put the latter to his mouth to cover a cough, and the coin slipped down. Recovered from the thoracic inlet. (Fig. 2.)

Age 6. A penny at level of top of sternum removed. (Fig. 3.)

PLATE X.

Fig. 1

Fig. 2

Fig. 3

Fig. 4

Fig. 5

Fig. 6

Fig. 7

Fig. 8

Fig. 9

Fig. 10

Fig. 11

Fig. 12

Fig. 13

Fig. 14

Fig. 15

Fig. 16

Fig. 17

Fig. 18

Fig. 19

Fig. 20

Fig. 21

Fig. 22

Fig. 23

*Half-penny.* Eighteen cases.

Age 1. Coin present two days, no symptoms. Radiogram shows it level with top of clavicle ; removed. (Fig. 1.)

Age 2. Coin swallowed same day, removed from level of suprasternal notch. (Fig. 4.)

Age 3. Coin just below cricoid, removed in 1 minute 40 seconds. (Fig. 5.)

Age 4. Coin at thoracic inlet ; removed.

Age 3. Coin at thoracic inlet ; removed. (Fig. 41.)

Age 4. Coin in centre of chest, found at 20·5 cm. from teeth ; removed. (Fig. 6.)

Age 4. Coin present 3 days, can take no solids. Skiagram shows it behind heart. Removed from behind left auricle at 25·5 cm. in 15 minutes. (Fig. 7.)

Age 7. Removed from thoracic inlet.

Age 3. Removed from just above sternum. (Fig. 8.)

Age 3. No symptoms. Coin swallowed same day, found behind cricoid and removed. (Fig. 9.)

Age 2. Top of coin level with bottom of cricoid ; removed.

Age 5. Coin removed from just below cricoid in 1 minute 30 seconds. (Fig. 10.)

Age 3. Removed from just above sternum. (Fig. 11.)

Age 4. Found at 12 cm. from teeth, removed in 1 minute 15 seconds. (Fig. 12.)

Age 6. Coin at 15 cm. from teeth, *i.e.* well in thoracic inlet. Removed in 2 minutes 12 seconds. (Fig. 13.)

Age 4. This coin I found 1 cm. below cricoid, *i.e.* at thoracic inlet. While demonstrating it, it was dislodged, and slipped down. It was recovered from just above the cardia ; time 16 minutes. (Fig. 14.)

Age 3. Coin two days in throat, no symptoms ; removed from level of thoracic inlet. (Fig. 15.)

Age 3. Removed from just above sternum. (Fig. 16.)

*Farthings.* Three cases.

Age 2. Coin swallowed two days before, can take fluids only; some dyspnœa. Coin removed from level of clavicle. (Fig. 17.)

Age 4. "He has swallowed a half-penny." A farthing was found at level of left bronchus, 5 cm. below thoracic inlet. Time 20 minutes. (Fig. 18.)

Age 4. "Was playing with his money-box and swallowed a farthing." Skiagram shows it level with top of sternum. On passing œsophagoscope edge of coin was seen and demonstrated —it showed particularly well. On removal it was found that forceps had grasped two exactly apposed farthings. (Fig. 20.)

*Bones.* Ten Cases.

Female, aged 43. A chicken bone swallowed the day before, removed from 35 cm. below the teeth. (Fig. 24.)

Female, aged 42. A rabbit bone lay in the left pyriform fossa, the point just visible by indirect laryngoscopy. (Fig. 25.)

Male, aged 33. Meat bone swallowed three days before; œdema of left pyriform fossa observed by indirect laryngoscopy. Bone removed from immediately beneath this point by direct œsophagoscopy. (Fig. 26.)

Female, aged 38. A rabbit bone lying behind the epiglottis, the sharp points causing much distress as they pricked the opposite walls of the pharynx every time the patient retched, which was frequently. (Fig. 27.)

Male, aged 11. This case is the tragedy of the series. While eating rabbit a bone stuck in his throat. His mother thrust her finger down and "cleared the throat." Next day he was brought to me looking very ill, unable to swallow at all, with great pain low in the neck, worse on attempting to swallow. The breath was fetid and the temperature 100·4° F. A radiogram failed to show any foreign body, but does show some commencing opacity of the tissues behind the top of the sternum (not noted before operation). I passed an œsophagoscope, and found immediately below the cricoideus a sharp splinter

### PLATE XI.

Fig. 24

Fig. 31

Fig. 28

Fig. 32

Fig. 25

Fig. 29

Fig. 26

Fig. 30

Fig. 33

Fig. 34

Fig. 27

Fig. 35

Fig. 36

Fig. 37

Fig. 38

Fig. 39

Fig. 40

(Note—Fig. 28 not to scale).

of bone (Fig. 28) sticking out of the left wall of the gullet, which was œdematous. The bone, which measured 2·2 cm., had penetrated the wall for about one-third of its length. On removing it, beads of pus began to escape. With punch forceps I opened an abscess into the lumen, and about a drachm of stinking pus welled up. Next day he could swallow fluids, but seemed ill, and on the third day there was definite broncho-pneumonia. An area of dullness could be percussed rather to the right of the mid-line in first and second intercostal spaces in front. The radiogram (Fig. 44) showed a large patch of opacity. He remained very ill, so ill that no further operation could be done, and had attacks of faintness followed by gulping up of fetid pus—the abscess was draining, though not properly. Subsequent radiograms showed a diminution of the shadow, but increasing lung changes. Getting steadily worse, he died on the seventh day from the accident. At autopsy we found an abscess cavity, fairly well defined, running down outside the left wall of the œsophagus, and ending among the great vessels, and severe broncho-pneumonia. Almost certainly the bone had been pushed through the œsophageal wall by well meaning but disastrous attempts to dislodge it. It is noteworthy that splinters of bone from soups and stews, that might be supposed to be sterile, always cause severe and immediate infection if they penetrate ; while pins and toys that are often not even reasonably clean tend to produce much less damage.

Female, aged 42. A mutton bone 17 cm. from the teeth, *i.e.* in thoracic inlet. (Fig. 29.)

The large bone in Figure 30 is included here to show what people will sometimes attempt to swallow ; it appears to have been lodged behind the cricoid, and was hawked up by the patient while preparations were being made to remove it.

Female, aged 54. A large fish bone, apparently from skull of haddock, at thoracic inlet. (Fig. 31.)

Male, aged 50. Fish bone embedded in the vallecula. (Fig. 32.)

Male, aged 33. Fish bone in base of tonsil, out of sight. (Fig. 34.) The difficulty in these two cases was more that of detecting the almost hair-like foreign body than of removing it.

Male, aged 28. Stout fish bone at thoracic inlet. (Fig. 33.)

*Meat.* Seven Cases.

Pieces of meat may be insufficiently chewed and so become impacted on swallowing being attempted ; or there may be some pathological condition of the œsophagus producing arrest of a normal bolus. The cases included here are those only in which the patient came " because something stuck."

Female, aged 59. A piece of meat stuck the day before ; a probang had been used, which pushed it down about an inch. The meat was firmly held by the lower part of the inferior constrictor, and on removal was found to be two inches long, the middle constricted as if a string had been tied round it.

Female, aged 54. Bolus of hard gristly meat impacted at œsophageal entrance.

Female, aged 48. Some meat had stuck the previous day, since when " everything she swallows comes up at once " ; pain behind the lower end of sternum. She has " always had a small swallow." A large piece of meat was found firmly impacted in the cardia ; so firmly that many fragments broke off when removal was attempted and the whole operation took nearly an hour.

Male, aged 73. Never any trouble in swallowing till a week ago some meat stuck. Since then he has been unable to swallow anything at all. The skiagram shows an apparently normal œsophagus with barium arrested in centre. At 19 cm. from teeth meat was found, occupying 7 cm. of the lumen. On removal, at 25 cm. was seen a small ulcer of the posterior wall, bleeding easily on touching. The lumen beyond was quite normal. Pathological report : carcinoma.

Female, aged 55. Meat stuck in throat one day. A bolus of meat was found just above the cardia, which was apparently

PLATE XII.

Fig. 41

Fig. 42

Fig. 43

Fig. 44

completely obstructed by malignant growth; no previous difficulty in swallowing. Gastrostomy performed.

Male, aged 55. He had previously been under me for carcinoma of the œsophagus, and after radium treatment was swallowing well. After several weeks he came up much worse, unable to take anything but liquids, and I naturally supposed the growth had recurred. On examination I found at the site of the growth, 22 cm. from teeth, a moderately firm fibrous stricture, covered with normal epithelium, and in it a small wedge of decomposing meat (confirmed by section). The stricture was dilated, without producing bleeding, and he is is doing well.

Male, aged 36. Subject of an old lye stricture, at 34·5 cm. A mass of meat occupied 9 cm. of the œsophagus above this.

*Dentures.* Four Cases.

Male, aged 33. A denture carrying two incisor teeth lodged at level of top of sternum. Under direct œsophagoscopy it was found that a metal hook was firmly fixed on the left wall of the œsophagus, the pin from which a third tooth had been broken being fixed in the right wall. A "double internal version" manœuvre was needed to disimpact the two hooks, the wall of the gullet being lifted away, and afterwards protected, by means of the lip of the œsophagoscope. At the moment when this had been accomplished, and before the denture could be seized, the patient vomited, and swallowed it down; it was passed *per vias naturales* four days later. (Fig. 21.)

Male, aged 49. A large upper denture impacted in the œsophageal entrance, and removed under general anæsthesia.

Female, aged 26. Denture in thoracic entrance. On attempting to seize it, it slipped down and was passed later.

Male, aged 48. A large upper denture lying in the hypopharynx for seventeen days, the upper part visible by indirect laryngoscopy. Removed under local anæsthesia. (Fig. 23.)

*Pins.* Four Cases.

Age 9 months. Baby noticed to have difficulty in swallowing, and pin then missed. No dyspnœa. Radiogram shows pin lying open, to right of mid-line, point upwards at level of hyoid. Removed by direct pharyngoscopy. (Fig. 35.)

Age 9 months. Swallowed a safety-pin thirteen days ago. Brought up with extreme dyspnœa, and much swelling of neck, and Mr. Angell James performed tracheotomy; three days later he removed the pin through an incision in the neck swelling, only the head of it being then in the lumen of the hypopharynx. Recovery.

Age 3 weeks. See page 113. (Fig. 37.)

Age 10. A drawing-pin sticking in the mucosa of the larynx, removed. (Fig. 36.)

*Toys.* Four Cases.

Age 1. Swallowed a "tin horse" two days before, can take fluids only. A small tin goat found at 13 cm. and removed. (Fig. 38.)

Age 6. A brass disc the size of a farthing removed from the level of the sterno-clavicular joint.

Age 11 months. Swallowed his tin watch two days before, now will not take anything. Watch removed from œsophageal entrance; ulceration of mucosa on either side where it had rested. Recovery. (Figs. 39 and 42.)

Age 3. Swallowed the key of a clockwork mouse. It lay with the two wings level with the top of the sternum, and the shank upward. Removed from this position under general anæsthesia, 45 seconds. (Fig. 40.)

## Figures and Tables

**Figure 45. f1:**
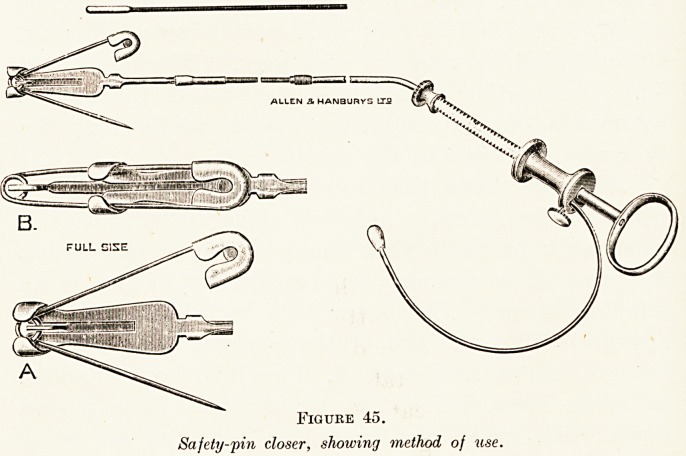


**Fig. 1 Fig. 2 Fig. 3 Fig. 4 Fig. 5 Fig. 6 Fig. 7 Fig. 8 Fig. 9 Fig. 10 Fig. 11 Fig. 12 Fig. 13 Fig. 14 Fig. 15 Fig. 16 Fig. 17 Fig. 18 Fig. 19 Fig. 20 Fig. 21 Fig. 22 Fig. 23 f2:**
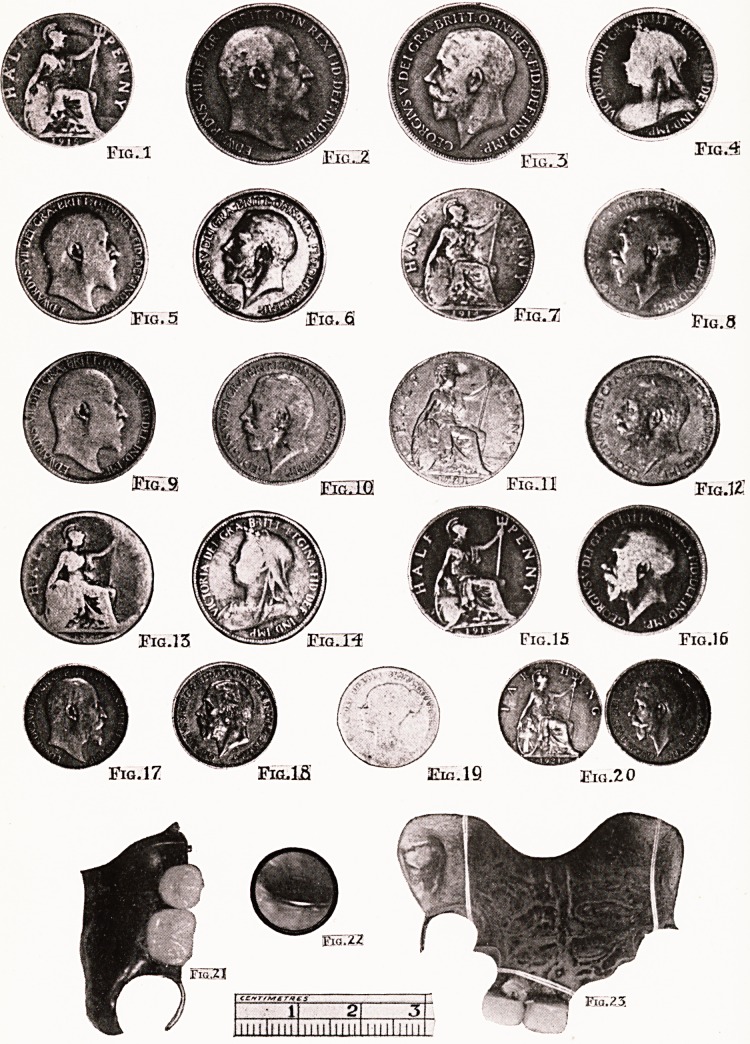


**Fig. 24 Fig. 25 Fig. 26 Fig. 27 Fig. 28 Fig. 29 Fig. 30 Fig. 31 Fig. 32 Fig. 33 Fig. 34 Fig. 35 Fig. 36 Fig. 37 Fig. 38 Fig. 39 Fig. 40 Fig. 28 f3:**
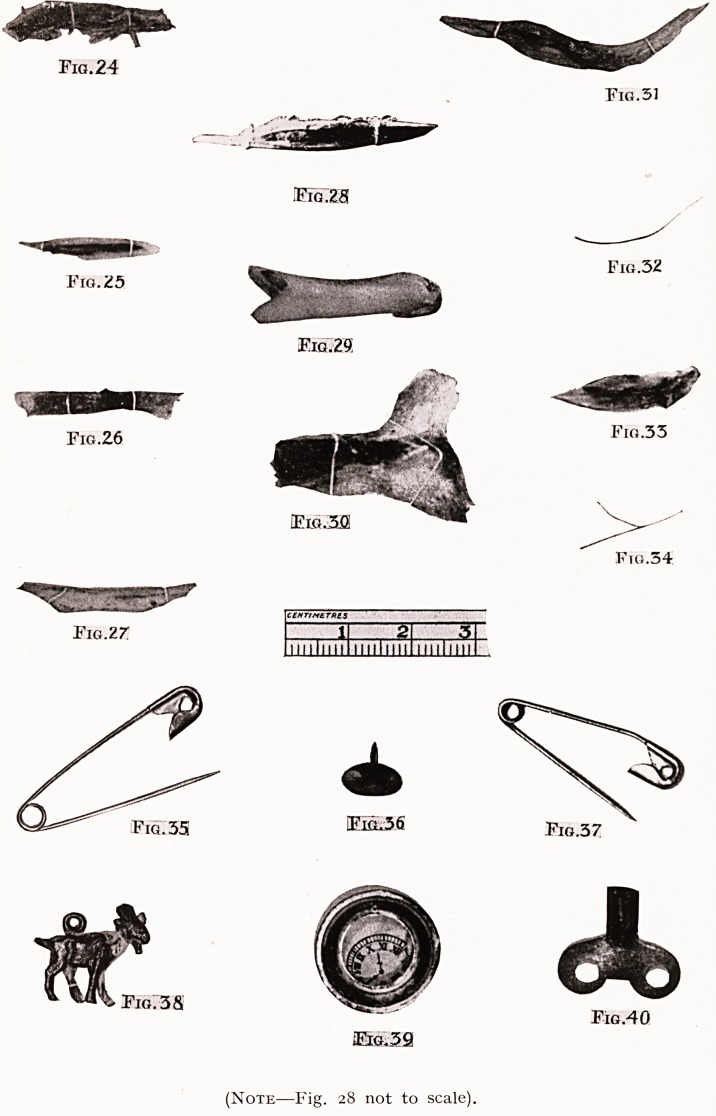


**Fig. 41 f4:**
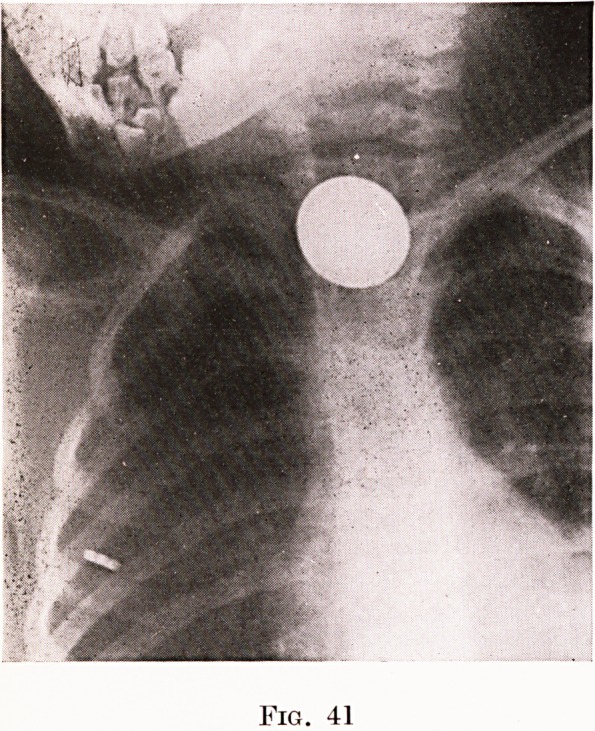


**Fig. 42 f5:**
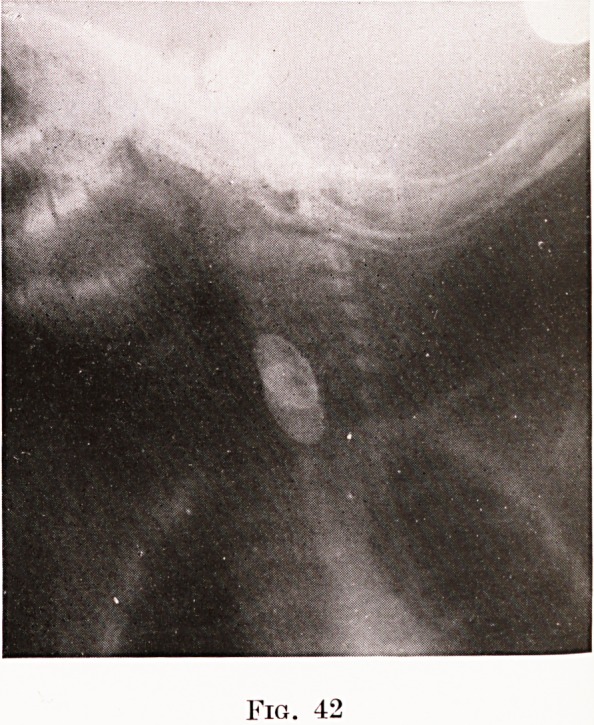


**Fig. 43 f6:**
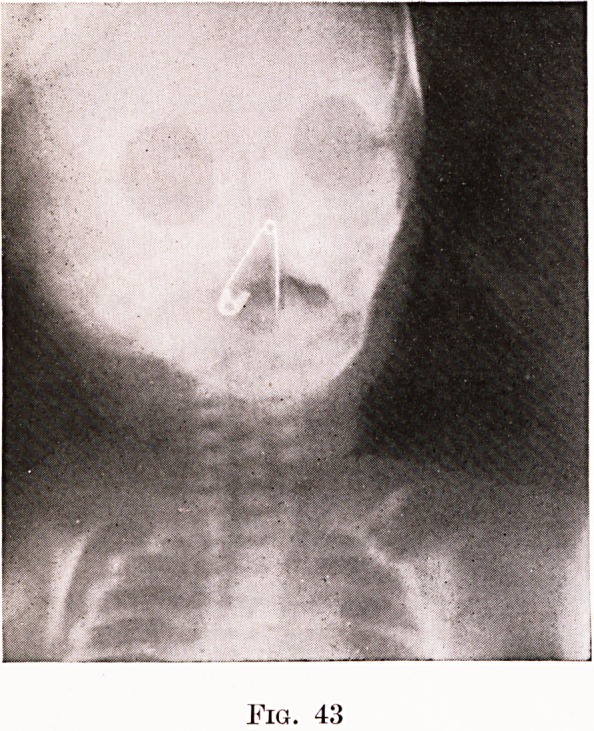


**Fig. 44 f7:**